# Supplementary material for: Symptoms and symptom clusters associated with SARS-CoV-2 infection in community-based populations: Results from a statewide epidemiological study
Source: PLoS One. 2021 Mar 24;16(3):e0241875. doi: 10.1371/journal.pone.0241875 (PMC7990210; doi:10.1371/journal.pone.0241875)
Supplement: S1 Table — Patients were considered to have a given combination if they reported one or more of the listed symptoms. Only combinations with a positive predictive value >20% were included. (DOCX) [file pone.0241875.s001.docx]

**S1 Table. Self-reported symptom combinations by participants undergoing SARS-CoV-2 testing. Patients were considered to have a given combination if they reported one or more of the listed symptoms. Only combinations with a positive predictive value >20% were included.**

| **Symptoms reported in the past 14 days** | **Number of individuals testing positive via RT-PCR  N (%)** | **Number of individuals testing negative via RT-PCR  N (%)** | **Sensitivity** | **Specificity** | **Positive Predictive Value** | **Negative Predictive Value** |
| --- | --- | --- | --- | --- | --- | --- |
| Overall Population Totals | 368 (4.6) | 7650 (95.4) |  |  |  |  |
| Loss of Smell (anosmia) OR Loss of Taste (ageusia) | 119 (32.3%) | 128 (1.7%) | 32.3% | 98.3% | 48.2% | 96.8% |
| Fever OR Loss of Smell (anosmia) | 170 (46.2%) | 205 (2.7%) | 46.2% | 97.3% | 45.3% | 97.4% |
| Fever OR Loss of Taste (ageusia) | 171 (46.5%) | 219 (2.9%) | 46.5% | 97.1% | 43.8% | 97.4% |
| Loss of Smell (anosmia) OR Vomiting | 101 (27.4%) | 132 (1.7%) | 27.4% | 98.3% | 43.3% | 96.5% |
| Loss of Taste (ageusia) OR Vomiting | 109 (29.6%) | 148 (1.9%) | 29.6% | 98.1% | 42.4% | 96.6% |
| Fever OR Vomiting | 131 (35.6%) | 182 (2.4%) | 35.6% | 97.6% | 41.9% | 96.9% |
| Chills OR Loss of Smell (anosmia) | 143 (38.9%) | 260 (3.4%) | 38.9% | 96.6% | 35.5% | 97.0% |
| Chills OR Loss of Taste (ageusia) | 144 (39.1%) | 267 (3.5%) | 39.1% | 96.5% | 35.0% | 97.0% |
| Chills OR Fever | 143 (38.9%) | 289 (3.8%) | 38.9% | 96.2% | 33.1% | 97.0% |
| Chest Pain OR Fever | 152 (41.3%) | 360 (4.7%) | 41.3% | 95.3% | 29.7% | 97.1% |
| Chest Pain OR Loss of Smell (anosmia) | 134 (36.4%) | 317 (4.2%) | 36.4% | 95.8% | 29.7% | 96.9% |
| Chest Pain OR Loss of Taste (ageusia) | 137 (37.2%) | 328 (4.3%) | 37.2% | 95.7% | 29.5% | 96.9% |
| Chills OR Vomiting | 86 (23.4%) | 228 (3.0%) | 23.4% | 97.0% | 27.4% | 96.3% |
| Chest Pain OR Chills | 115 (31.2%) | 397 (5.2%) | 31.2% | 94.8% | 22.5% | 96.6% |
| Fever OR Shortness of Breath | 150 (40.8%) | 537 (7.1%) | 40.8% | 92.9% | 21.8% | 97.0% |
| Loss of Taste (ageusia) OR Shortness of Breath | 140 (38.0%) | 511 (6.7%) | 38.0% | 93.3% | 21.5% | 96.9% |
| Loss of Smell (anosmia) OR Shortness of Breath | 133 (36.1%) | 493 (6.5%) | 36.1% | 93.5% | 21.2% | 96.8% |
| Fever OR Muscle Ache (myalgia) | 168 (45.7%) | 645 (8.5%) | 45.7% | 91.5% | 20.7% | 97.2% |
| Loss of Smell (anosmia) OR Muscle Ache (myalgia) | 160 (43.5%) | 613 (8.1%) | 43.5% | 91.9% | 20.7% | 97.1% |
| Diarrhea OR Fever | 151 (41.0%) | 583 (7.7%) | 41.0% | 92.3% | 20.6% | 97.0% |
| Loss of Taste (ageusia) OR Muscle Ache (myalgia) | 159 (43.2%) | 621 (8.2%) | 43.2% | 91.8% | 20.4% | 97.1% |
| Fever OR Loss of Smell (anosmia) OR Loss of Taste (ageusia) | 177 (48.1%) | 242 (3.2%) | 48.1% | 96.8% | 42.2% | 97.5% |
| Fever OR Loss of Smell (anosmia) OR Vomiting | 172 (46.7%) | 243 (3.2%) | 46.7% | 96.8% | 41.4% | 97.4% |
| Loss of Smell (anosmia) OR Loss of Taste (ageusia) OR Vomiting | 123 (33.4%) | 175 (2.3%) | 33.4% | 97.7% | 41.3% | 96.8% |
| Fever OR Loss of Taste (ageusia) OR Vomiting | 173 (47.0%) | 257 (3.4%) | 47.0% | 96.6% | 40.2% | 97.4% |
| Chills OR Loss of Smell (anosmia) OR Loss of Taste (ageusia) | 156 (42.4%) | 293 (3.9%) | 42.4% | 96.1% | 34.7% | 97.2% |
| Chills OR Fever OR Loss of Smell (anosmia) | 178 (48.4%) | 341 (4.5%) | 48.4% | 95.5% | 34.3% | 97.5% |
| Chills OR Fever OR Loss of Taste (ageusia) | 179 (48.6%) | 351 (4.6%) | 48.6% | 95.4% | 33.8% | 97.5% |
| Chills OR Loss of Smell (anosmia) OR Vomiting | 147 (39.9%) | 290 (3.8%) | 39.9% | 96.2% | 33.6% | 97.1% |
| Chills OR Loss of Taste (ageusia) OR Vomiting | 146 (39.7%) | 297 (3.9%) | 39.7% | 96.1% | 33.0% | 97.1% |
| Chills OR Fever OR Vomiting | 144 (39.1%) | 318 (4.2%) | 39.1% | 95.8% | 31.2% | 97.0% |
| Chest Pain OR Fever OR Loss of Smell (anosmia) | 185 (50.3%) | 410 (5.4%) | 50.3% | 94.6% | 31.1% | 97.5% |
| Chest Pain OR Fever OR Loss of Taste (ageusia) | 186 (50.5%) | 422 (5.6%) | 50.5% | 94.4% | 30.6% | 97.5% |
| Chest Pain OR Loss of Smell (anosmia) OR Loss of Taste (ageusia) | 149 (40.5%) | 351 (4.6%) | 40.5% | 95.4% | 29.8% | 97.1% |
| Chest Pain OR Fever OR Vomiting | 153 (41.6%) | 396 (5.2%) | 41.6% | 94.8% | 27.9% | 97.1% |
| Chest Pain OR Loss of Smell (anosmia) OR Vomiting | 135 (36.7%) | 358 (4.7%) | 36.7% | 95.3% | 27.4% | 96.9% |
| Chest Pain OR Loss of Taste (ageusia) OR Vomiting | 138 (37.5%) | 369 (4.9%) | 37.5% | 95.1% | 27.2% | 96.9% |
| Chest Pain OR Chills OR Loss of Smell (anosmia) | 166 (45.1%) | 449 (5.9%) | 45.1% | 94.1% | 27.0% | 97.3% |
| Chest Pain OR Chills OR Loss of Taste (ageusia) | 163 (44.3%) | 456 (6.0%) | 44.3% | 94.0% | 26.3% | 97.2% |
| Chest Pain OR Chills OR Fever | 163 (44.3%) | 473 (6.2%) | 44.3% | 93.8% | 25.6% | 97.2% |
| Fever OR Loss of Smell (anosmia) OR Shortness of Breath | 184 (50.0%) | 580 (7.6%) | 50.0% | 92.4% | 24.1% | 97.4% |
