# Supplementary material for: Symptoms and symptom clusters associated with SARS-CoV-2 infection in community-based populations: Results from a statewide epidemiological study
Source: PLoS One. 2021 Mar 24;16(3):e0241875. doi: 10.1371/journal.pone.0241875 (PMC7990210; doi:10.1371/journal.pone.0241875)
Supplement: Attachment — Submitted filename: PONE Response to Reviewers - FINAL - 20210131.docx [file pone.0241875.s002.DOCX]

**Dear Editors,**

Thank you for the opportunity to further revise our manuscript (PONE-D-20-33594) entitled *"Symptoms and symptom clusters associated with SARS-CoV-2 infection in community-based populations: Results from a statewide epidemiological study."*

Below, we document how we have addressed each issue raised by the reviewers on a point-by-point basis. We also acknowledge the comments of the Associate Editor. Overall, we greatly appreciate the enthusiasm for our work and for the enriching comments we have received.

We believe the manuscript is strengthened by the suggestions and feedback. We genuinely appreciate the opportunity to have our work considered by *PLOS One*.

Sincerely,

Brian Dixon, PhD
Corresponding Author

| Reviewer #1 | Authors’ Response |
| --- | --- |
| Introduction: -Update global cases and deaths as they have surpassed 54 million & 1.3 million as of Nov. 16 | We agree these figures should be updated. We therefore updated the figures as of January 31, 2021 prior to re-submission to PONE.  The new figures read as follows:  “Globally there have been more than 102 million cases and over 2.2 million deaths as of January 31, 2021.” |
| Introduction: -End of first paragraph will need to be reworked since fall/winter is here and COVID is spiking again. | Agree that given the length of the review cycle these sentences should be revised. The revised sentences now read as follows:  “Most recently, cases in the U.S. as well as Europe surged heading into the winter holidays, with many nations re-implementing mitigation techniques to slow the spread of the virus.” |
| Introduction: -Paragraph 2, calling COVID-19 an “influenza-like illness” could be confusing given the misconception among some that it is the same virus as flu or has the same severity of flu. | We do not wish to confuse readers or give conspiracy theorists ammunition that COVID is the same as the flu. Therefore, we have updated the language as follows:  “…influenza and other respiratory diseases.” |
| Introduction: -Clarify that dyspnea is shortness of breath when shortness of breath first mentioned like is done with loss of taste (ageusia) and smell (anosmia). | Good suggestion. We have updated the second sentence in the second paragraph of the Introduction as follows:  “…shortness of breath (dyspnea).” |
| Introduction: -Anything updated about cardiovascular effects or are those not symptoms that are identified outside of a healthcare setting? | These questions from the reviewer are not clear given that cardiovascular effects signal end organ involvement following disease as opposed to a clinical sign/symptom. The most prominent symptoms of cardiovascular involvement would be dyspnea and chest pain, both of which are included as symptoms asked of participants in this study.  We did perform a quick scan of the literature and confirmed that CV effects are not described as being predictive of COVID-19. Further, we wish to highlight that the Cochrane review we cite in the last paragraph of the Introduction concluded this about cardiovascular symptoms: “Gastrointestinal symptoms and cardiovascular features…have very little diagnostic value.” Therefore our findings are consistent with the conclusions from the review.  Given this paper is focused on clinical diagnosis, the two major CV effect symptoms are included, and prior studies find similar implications for the symptoms associated with CV effects, we did not update the introduction. |
| Methods: -Were the participants mentioned who were not randomly selected and sought testing in open-testing sites the same participants who were recruited by civic leaders in African American and Hispanic communities? First paragraph of methods mentions random selection, those seeking open-testing sites and nonrandom testing of underrepresented groups. Clarify if 2 are the same. | Yes – the individuals who sought open testing were tested at the same sites recruited by civic leaders in minority communities. Since those sites were open, some individuals at those sites were not specifically recruited by leaders. They may have simply noticed the signs/messages and came in for testing that day.  We have clarified in the manuscript as follows:  “Each wave included two groups: individuals that were randomly selected for invitation-only testing, and individuals from predominantly minority communities encouraged to attend open-testing at sites throughout the state.” |
| Methods: -For groups that were randomly selected, were they asked to be tested? How was that testing accessed and tracked? | Randomly selected individuals were asked to make an appointment for testing at one of several invitation-only sites operated by the state department of health on specific dates during each testing wave. Individuals selected based on tax records were mailed postcards and received a phone call and text message from the state, informing them of their selection and encouraging them to come out for testing.  The testing at the closed sites was distinguished by the state health department from the open sites based on the location of the sample collection. Site of collection was made available to the study team for analysis.  We have clarified this in the manuscript as follows:  “Randomly selected individuals (N=15,495) received a postcard, text message, and phone call inviting them for COVID-19 testing at specific sites set-up across the state by the department of health.”  “Data collected from participants, along with laboratory test results, were captured separately by the state health department and distinguished from other testing sites for the study team.” |
| Results: -With the findings being so different between the random and nonrandom sample, it is difficult to interpret | Table 1 is provided as a reference to distinguish between the random and nonrandom participants. The table highlights that nonrandom participants were more likely to be racial or ethnic minorities. The final column indicates that, like other studies, minority groups were more likely to be positive for infection.  The remaining tables and figures focus on the pooled analysis of symptoms among those tested, regardless of testing site. Those results focus on which symptoms were associated with active infection with SARS-CoV-2 virus. Because all participants were asked to report symptoms, and a sensitivity analysis we performed using race, age, and gender did not find anything different from the pooled analysis, we are confident that our results can be interpreted regardless of testing site. |
| Reviewer #2 | Authors’ Response |
| Methods- Study Participants and Recruitment- How many people were randomly selected and how many in the group that presented on their own (is this the same group that was recruited or is this another distinction)? If they are the same then the terminology should be consistent- random selection vs recruitment (instead of switching to "open community testing" in Table 1). If the groups are not same, Were the characteristics of these groups compared statistically? .How was the randomization undertaken? | In response we made several edits to the Methods section to clarify the two groups: randomly selected individuals and nonrandom individuals recruited from predominantly minority communities. We chose not to use the term “recruited” or “recruitment” for the second group because technically both groups were recruited to participate in the study, but how we reached each population was different.  We randomly selected 15,495 individuals across the state using tax records. We added this number to the paper.  The randomization used multiple years of tax records to identify individuals who resided in the state. Each member of a household, including dependents, were randomized for selection. One member from each household was selected for invitation. These details are available in the MMWR publication as well as a forthcoming PNAS paper that details our methodology. |
| Testing- How were the samples distributed to the two labs? Any difference in viral load detection ability in the two labs? Were random samples sent to one lab and recruited to the other? | Thank you for these questions.   1) Samples were distributed to the two labs irrespective of the collection site and based on date, lab capacity, etc. 2) We did not measure viral load. There might have been differences in viral load detection between the two labs. However, a difference in some samples would not impact the results from a large population study. |
| Results Table 1-Was the percent of positive tests different in the random group vs the recruited group? | Yes, as expected, the recruited (more vulnerable) group possessed a higher % positive. This was reported in our MMWR paper on the overall results from Wave 1. Racial and ethnic minorities were more likely to test positive, and because the recruited participants came from those communities we observed a higher overall % positive from the recruited versus the randomly selected individuals. |
| For Table 2- calculating negative predictive value of symptom combinations - Does this mean all three were absent or one of the symptoms was absent, to be considered negative? | As presented in Table 2, all symptoms must be present to be considered positive. If any of the symptoms were missing then the symptom combination was considered negative. |
| The authors should also present the data with sensitivity for symptoms groupers with "or" function to demonstrate that if certain group of symptoms are all absent the sensitivity is higher. Thus can show that a screening list of certain symptoms will work well to screen individuals at workplaces and community settings (similar to the CDC list). Although not required, it will definitely make the study even more interesting. | We appreciate this suggestion from the reviewer. When examining symptom groups using an OR operator, where patients meet inclusion if any one symptom is present, sensitivities increase while PPV decreases (as one would expect). Therefore, symptom groups based on OR would not be better for clinical decision-making as they are less specific and possess lower PPV.  Although the symptom groups using OR do not perform better diagnostically, we do want our paper to be more interesting. Therefore, we created a table with the OR-based symptom groups and include it as Table S1 to the paper. If the Editors wish the table to be included in the main paper, we are open to that. However, we felt it would be best as supplemental info. |
| For Table 3- Those presenting on their own are more likely to have had symptoms with higher positivity. These were also predominantly ethnic minorities. The higher odds ratio in Hispanic patients may be strongly influenced by this fact and should be interpreted with caution. The authors have mentioned this in the limitations. | We agree, which is why we added the caveat to the Limitations section. |
| Discussion Second para- "These symptoms are highly predictive for positive status" should read "These symptoms individually are highly specific for COVID and in combination with fever are highly predictive for positive status". | Thank you for this suggestion. We have reworded the sentence as follows, per your suggestion:  “These symptoms individually are highly specific for COVID-19 infection and, in combination with fever, are highly predictive of positive status.” |
